# Audit of amylase and lipase requests in suspected acute pancreatitis and cost implications, South Africa

**DOI:** 10.4102/ajlm.v11i1.1834

**Published:** 2022-09-26

**Authors:** Annie E. Cook, Thumeka P. Jalavu, Annalise E. Zemlin

**Affiliations:** 1 Department of Clinical Biochemistry, Derriford Combined Laboratory, University Hospitals Plymouth National Health Service Trust, Derriford Hospital, Plymouth, United Kingdom; 2 Division of Chemical Pathology, Department of Pathology, Stellenbosch University and National Health Laboratory Service, Tygerberg Hospital, Cape Town, South Africa

**Keywords:** audit, pancreatitis, amylase, lipase, cost

## Abstract

**Background:**

The internationally accepted criteria for the diagnosis of acute pancreatitis (AP) requires two of the three following features to be present: characteristic abdominal pain, elevated serum amylase and/or lipase enzymes, or consistent imaging results. However, sensitivity and specificity can vary depending on the population and cut-off values used.

**Objective:**

This study evaluated the suitability of amylase and lipase as first-line diagnostic biomarkers of suspected AP for the local population served by Tygerberg Hospital, South Africa.

**Methods:**

This retrospective analysis was conducted in June 2019 using all amylase and/or lipase request data from December 2018. Patient clinical data were included in sensitivity and specificity analyses of amylase, lipase or dual requests for diagnosis of AP. Cost per test data were obtained from the National Health Laboratory Service and used to calculate the total cost of the tests and potential savings.

**Results:**

Sensitivity for lipase was 90.0% compared to 50.0% for amylase. Specificity was similar for singular measurements of lipase and amylase. Dual measurement of amylase and lipase showed no improvement in sensitivity (83.3%) and only a minor increase in specificity (97.4%) compared with measurement of lipase alone. The estimated savings was R2522.85 ($174.98 USD), with a potential annual cost saving of R84 423.74 ($5855.69 USD).

**Conclusion:**

Lipase was shown to be a more sensitive biomarker compared to amylase for the screening of AP, providing evidence for laboratories to educate local staff and promote improved requesting practices by clinicians. Additionally, preventing unnecessary dual requests may reduce costs.

## Introduction

The Atlanta Symposium in 1992 sought to provide a unified international consensus for the classification of acute pancreatitis (AP). The revised version of the Atlanta criteria expands on the previous definition to include a classification of severity, defines imaging morphology and discriminates AP as either intestinal oedematous pancreatitis or necrotising pancreatitis.^1^ According to the revised Atlanta criteria and several other international guidelines, AP diagnosis requires two of the following three criteria to be present: the rapid onset of severe and persistent epigastric pain consistent with AP; serum lipase and/or amylase levels greater than three times the upper limit of normal (ULN); or imaging consistent with AP, using contrast-enhanced computed tomography, magnetic resonance imaging or transabdominal ultrasonography.^1,2,3,4^

The gold standard for diagnosis of AP is often considered to be supporting radiological evidence,^5^ however, this option is rarely considered suitable as a first-line test, and therefore pancreatic enzyme biomarkers provide an essential criterion for quick and accurate assessment in cases where AP is suspected. Currently serum amylase and lipase remain the most commonly requested tests for investigation of AP, which is supported by several international guidelines.^1,3,4^ The limitations of these biomarkers is that both lipase and amylase can also be raised to levels of greater than three times the ULN in non-pancreatic conditions, such as renal disease, appendicitis and cholecystitis.^6^ Liraglutide, a glucagon-like peptide-1 receptor agonist used for the treatment of diabetes, has also been shown to falsely elevate serum amylase and lipase levels.^7^ Additionally, neither enzyme can determine aetiology or severity of AP in adults.^8^ Despite this, lipase is considered to be the preferential diagnostic biomarker.^9^ This was first suggested in United Kingdom guidelines 2005,^4^ due to the longer half-life of lipase compared with amylase; such that lipase still remains detectable 7–14 days after symptom onset, compared with 3–4 days with amylase.^6^ Lipase is now globally accepted as the superior analyte for the investigation of AP, with many international studies and reviews evidencing its improved sensitivity and specificity compared with that of amylase.^5,10,11^

Sensitivity and specificity can vary depending on the population included and the cut-off values used. The two most common aetiologies of AP internationally are gallstones or alcohol abuse. The most common cause of AP in the Tygerberg community where this study was performed is alcohol abuse.^11,12^

We aimed to determine the relative sensitivity and specificity of lipase compared with amylase for the diagnosis of AP within the local population served by Tygerberg Hospital for the month of December 2018, and to determine what proportion of these requests were clinically indicated. The recommended test for AP is serum lipase, where available; amylase is used in settings where serum lipase is not available. The practice of requesting both tests (dual testing) in the initial evaluation is not recommended but is seen on a regular basis in our laboratory. This practice is not cost effective; it is therefore important to assess how prevalent it is so that it can be addressed. The final aim, therefore, was to review the absolute numbers of dual versus single amylase and lipase requests for the whole of 2018 and to calculate the potential cost-savings associated through changes in requesting practices since lipase became available in our laboratory.

## Methods

### Ethical considerations

Ethical approval was obtained to access the clinical data of patients where these analytes had been requested. This was sought in advance of the project start and was granted by the Health Research Ethics Committee of the Faculty of Medicine and Health Sciences at Stellenbosch University (reference number: N19/03/036). Patient consent was not obtained as a waiver of consent was awarded by the approving ethics committee. The clinical records of patients were only accessed if they were included in the study and were only accessed by authors of this paper. Full anonymity of patients was maintained throughout the study and all data was stored in password protected files.

### Study site

This was a retrospective clinical audit of amylase and lipase requesting at Tygerberg Hospital, Cape Town, South Africa. Tygerberg Hospital is a tertiary hospital in Parow, Cape Town and provides inpatient and outpatient care to public health-sector users. It is a 1400 bed multidisciplinary teaching hospital affiliated with Stellenbosch University. The National Health Laboratory Service (NHLS) Chemical Pathology laboratory at Tygerberg Hospital provides 24-h diagnostic service. The NHLS is the preferred provider of pathology services to the public health sector.

### Data collection

Pathology information technology provided an anonymised data set for all amylase and lipase requests for 2018 that included the following parameters: patient hospital numbers, ward location, date of birth and the amylase and/or lipase results. Data from 1 December 2018 to 31 December 2018 were chosen as an initial subset for analysis and the clinical notes surrounding the dates of the amylase and lipase requests within this month were then individually scrutinised. Enterprise Content Management software (Open Text ECM, OpenText Corporation, Waterloo, Ontario, Canada), a web-based electronic patient management system, was used to assess each patient record and to determine whether any consistent imaging was performed, the patient clinical symptoms upon presentation and the final patient diagnosis. A diagnosis of AP was considered confirmed if two of the three Atlanta criteria were fulfilled or in most cases, by an explicit statement provided in the clinical notes. Requests were only included and analysed where the patient was being managed at Tygerberg Hospital and the laboratory test(s) had been carried out at the Tygerberg NHLS laboratory. Records were excluded if patients were aged < 13 years old, had a recent history of penetrating or blunt abdominal trauma and where notes were incomplete or unclear. The cost per test data were obtained from the NHLS and used to calculate the total cost of the tests over the month of December and potential savings if dual testing were to be eliminated.

### Laboratory analyses

Samples were analysed for amylase and lipase using an enzymatic colorimetric methodology and performed on the Roche^®^ cobas^®^ 6000 analyser (Roche Diagnostics, Mannheim, Germany) at the Tygerberg Hospital NHLS laboratory. Tygerberg Hospital is accredited by the South African National Accreditation System that regularly participates in external quality assessment to retain this status.

### Statistical analysis

All data used within the study were recorded, sorted and analysed using Microsoft Excel (Microsoft Corporation, 2019 [16.0], Redmond, Washington, United States). The normal ranges used by Tygerberg Hospital at the time this audit was conducted were 20 U/L – 104 U/L for serum amylase and 13 U/L – 60 U/L for lipase. Based on these ranges, patient results for all amylase and/or lipase results for December 2018 were either designated ‘≤ 3ULN’ less than or equal to three times the ULN; or ‘> 3ULN’, if the result was greater than three times the ULN. Presenting clinical details (if available), final diagnosis and relevant imaging were also recorded and then allocated either ‘Yes’, ‘No’ or ‘Unknown’ depending on whether they were considered consistent with AP. Based on these parameters, amylase and/or lipase were designated either a false positive (FP), false negative (FN), true positive (TP) or true negative (TN) status. The relative sensitivity, specificity, positive predictive value (PPV) and negative predictive value (NPV) were calculated using the following equations:


$$\begin{array}{l} \text{Sensitivity}=\text{TP}/(\text{TP}+\text{FN})\\ \text{Specificity}=\text{TN}/(\text{TN}+\text{FP})\\ \text{PPV}=\text{TP}/(\text{TP}+\text{FP})\\ \text{NPV}=\text{TN}/(\text{TN}+\text{FN}) \end{array}$$ [Eqn 1]

Microsoft Excel was also used to provide estimated cost savings associated with changes in amylase- and lipase-requesting practices. The sample numbers obtained from the 1 to 31 December dataset were used to extrapolate predicted annual savings.

## Results

A total of 268 patients had either a lipase or amylase test (or both) carried out during December 2018 at Tygerberg Hospital (Table 1). Forty-six of the 268 requests were excluded from the final data analysis, including duplicate requests (*n* = 16), requests on patients where notes were incomplete or unclear (*n* = 10) , patients with a recent history of penetrating or blunt abdominal trauma (*n* = 9), patients with chronic pancreatitis (*n* = 9), and patients < 13 years old (*n* = 2). A total of 222 patients were therefore included in the final data analysis; 12 (5.4%) patients had a final diagnosis of AP.

**TABLE 1:** Amylase and lipase results for acute pancreatitis testing obtained from Tygerberg Hospital, South Africa, 1–31 December 2018.

Diagnosis for acute pancreatitis	Test positive for AP (> 3ULN)		Test negative for AP (≤ 3ULN)	
	Amylase	Lipase	Amylase	Lipase
Positive	1 (TP)	9 (TP)	1 (FN)	1 (FN)
Negative	1 (FP)	6 (FP)	68 (TN)	190 (TN)

3ULN, three times the upper limit of normal; AP, acute pancreatitis; FN, false negative; FP, false positive; TN, true negative; TP, true positive.

Specificities were relatively equivocal for single amylase (98.6%) and lipase tests (96.9%) (Table 2). Sensitivity of amylase showed a significant difference, however, at only 50.0% compared to lipase at 90.0% for the subset of data analysed. Dual requesting showed an equivocal specificity of 97.4%, however the sensitivity was calculated to be 83.3%, which was 6.7% less than lipase testing alone.

**TABLE 2:** Sensitivity, specificity, positive predictive value, negative predictive value and respective 95% confidence interval for amylase, lipase and dual testing for acute pancreatitis at Tygerberg Hospital, 1–31 December 2018.

Statistic	Amylase		Lipase		Dual testing	
	Value %	95% CI†	Value %	95% CI	Value %	95% CI
Sensitivity	50.0	1.3–98.7	90.0	55.5–99.8	83.3	51.6–97.9
Specificity	98.6	92.2–100.0	96.9	93.5–98.9	97.4	94.6–98.9
PPV	50.0	8.4–91.6	60.0	39.9–77.2	58.8	39.7–75.6
NPV	98.5	94.4–99.6	99.5	96.7–99.9	99.2	97.3–99.8

CI, confidence interval; NPV, negative predictive value; PPV, positive predictive value.

† , Confidence intervals obtained using the ‘exact’ Clopper-Pearson method.

In December 2018, lipase was requested singly 151 times compared with amylase, which was requested 16 times (Table 3). Fifty-five of the 222 patients (25%) had dual testing for amylase and lipase. The price of amylase and lipase tests was R45.87 (South African rand [ZAR], equivalent to $3.10 United States dollars [USD]) per individual test for the period of December 2018. The cost of dual requests for the month of December was R2522.85 ($174.98 USD); that of unnecessary tests was R733.92 ($50.90 USD) and for those not clinically indicated, R5320.92 ($369.06 USD). The projected potential annual cost saving from all these additional or unnecessary tests is R84 423.74 ($5855.69 USD) (Table 3).

**TABLE 3:** Amylase and lipase test requests and predictive cost savings, Tygerberg Hospital, 1–31 December 2018.

Potential savings	1–31 December 2018 (*N*)	1–31 December 2018 cost savings†		Annual numbers	Per annum saving for 2018	
		ZAR	USD		ZAR	USD
Patient requests for amylase and/or lipase	222	-	-	4209	-	-
Lipase-only requests	151	-	-	3318	-	-
Amylase-only requests	16	-	-	378	-	-
Dual requests for amylase and lipase	55	R2522.85	$174.98	513	R11 765.66	$816.06
Unnecessary duplicated requests	16	R733.92	$50.90	192	R8807.04	$610.85
Requests not clinically indicated	116	R5320.92	$369.06	1392	R63 851.04	$4428.68
Total potential savings	-	-	-	-	R84 423.74	$5855.69

R, South African Rand (ZAR); $, United States dollars (USD)

† , Estimated savings based on 1–31 December 2018 data subset; based on a single amylase or lipase test costing R45.87 (ZAR, $3.20 USD [December 2018]).

## Discussion

The aim of this study was to compare the relative sensitivities and specificities of lipase and amylase for the diagnosis of AP across the Tygerberg Hospital population, and to evaluate this in the context of current requesting practices. The initial hypothesis by the laboratory was that amylase was often requested preferentially to that of lipase; the results of this audit, however, have shown that the opposite is true and that most requests in 2018 were, instead, for lipase. Additionally, we examined the cost implications associated with current requesting practices and have estimated significant cost-saving potential with a change to single lipase requesting in place of unnecessary dual requests.

Lipase and amylase have been shown to have high specificity with respect to the diagnosis of AP,^5,10,11^ when used at the levels of greater than three times the ULN, as recommended in the Atlanta criteria.^2^ In this context, specificity is the ability of these biomarkers to correctly identify patients without AP when they are at levels of less than three times ULN as stipulated in the Atlanta criteria.^2^ Whilst both biomarkers showed similar specificities from the results of this study, at 98.6% for amylase and 96.9% for lipase, sensitivity between the two analytes differed significantly, which was likely to have been significantly impacted by the small sample size. Despite this, the results of this study agreed with the widely accepted international consensus, that lipase is a superior biomarker in cases of suspected AP, with a sensitivity of 90% compared with only 50% for amylase.

This study was useful because it provided data based on the local Tygerberg Hospital population. It is widely acknowledged that the aetiology of AP can have a significant impact on the serum levels of these pancreatic enzymes,^11^ thus it can provide more relevant data for the local medical community to guide recommendations on appropriate requesting practices. Amongst the Tygerberg population, alcohol abuse is the primary cause of AP,^11,12^ which typically exhibits lower levels of amylase and lipase in comparison to the next most common cause of AP, gallstones. This could impact the relative sensitivity and specificity of these analytes according to the levels stipulated in the Atlanta criteria.^1^

The sensitivity and specificity from dual requests showed minimal improvement in specificity when compared to lipase alone, and a reduced sensitivity. There were 513 dual requests for 2018. This represents around 257 unnecessary test requests annually which can be costly and potentially detrimental with respect to reduced sensitivity.

A recent 2017 review of nine studies by Ismail and Bhayana,^8^ which included studies carried out by Chang and Chung^5^ and Hofmeyr et al.,^11^ concluded a negligible difference in sensitivity and specificity from dual testing of lipase and amylase compared with singular testing of lipase.^5,8,11^ Whilst studies by Basnayake and Ratnam,^9^ and Ismail and Bhayana^8^ have commented that the ratio between amylase and lipase levels could help direct clinicians to the aetiology of AP,^8,9^ it was generally concluded across several reviews and studies, that dual testing was not a cost-effective option for diagnosis of AP.^5,8,11,13^

A prospective study by Hofmeyr et al.^11^ concluded that on admission to Tygerberg Hospital lipase sensitivity was significantly improved at 91% compared with 62% for amylase. The specificity of amylase and lipase testing for this population group were comparable, at 93% and 92%, respectively. The recommendations from this study were to promote lipase as the biomarker of choice locally for the diagnosis of AP.^11^

A potential strategy for overcoming inappropriate dual requesting and the use of amylase instead of lipase in these patient groups could be to limit or introduce a local procedure for demand management of amylase requests. The largest potential cost-saving identified from this study, however, would be through the promotion of greater clinically led requesting in suspected AP. Over half of the requests included within this study were considered inappropriate based on the clinical details and final diagnosis provided in the patient notes.

### Limitations

There were several important limitations that should be considered with respect to this study. Firstly, and most significantly, only 12 of the 222 patients used for the final data analysis had confirmed AP. Despite this small subset of data, sensitivity and specificity of lipase and amylase was consistent with a previous study of these biomarkers within the Tygerberg population.^11^ However, it should be noted that the large sensitivity differences observed between amylase and lipase are likely because of the small sample size used. The second limitation of this study is that requests were only considered clinically relevant if the patient notes mentioned abdominal, epigastric or associated pain or overtly queried AP. It is important to note that there are other disease states where these biomarkers may be requested for use in diagnosis and monitoring, where the patient may not typically present with abdominal pain for example, pancreatic cancer, mumps and cystic fibrosis.^14,15^ Whilst these test requests would be considered clinically relevant, for the purposes of this audit, these cases were not accounted for in the final data and cost analysis. In addition, it is important to note that the main AP aetiology will vary between different population groups and this study only includes the local Tygerberg population. Another consideration that has not been accounted for as part of this study but may affect the associated sensitivities and specificities of these analytes is assay performance across different analytical platforms and methods.

Notwithstanding the limitations of this study, even this small dataset supports the existing literature, that lipase shows improved sensitivity when compared with amylase for the diagnosis of AP. Ideally, a repeat clinical audit should be conducted to include at least a year of data which would enhance the robustness of these results. Indeed, a comparative clinical audit from a United Kingdom population, where the most common aetiology of AP differs, would help to demonstrate that lipase is a better marker, regardless of aetiology.

The results of this study have helped to better equip the laboratory to inform and promote more clinically led and evidence-based requesting practices amongst local clinicians. Whilst these pancreatic enzyme biomarkers can provide an important tool for clinicians trying to detect or eliminate an AP diagnosis, it is also important for requestors to have a full understanding of the potential limitations associated with them. A prompt diagnosis of AP is important for limiting associated complications if left undetected and ultimately improving patient outcomes.

### Conclusion

The results of this clinical audit support a growing body of evidence that lipase is superior as a first-line test for suspected AP and that there is little additional clinical value derived from dual requesting of lipase and amylase. Despite the small subset of data used within this audit, we have shown that the sensitivity and specificity of lipase and amylase was consistent with a previous study of these biomarkers within the Tygerberg population, and that lipase is the superior biomarker in terms of sensitivity. It is therefore recommended that dual requests for amylase and lipase are replaced by singular lipase requests where AP is suspected.
